# Supplementary figures and images for: FEM: mining biological meaning from cell level in single-cell RNA sequencing data
Source: PeerJ. 2021 Nov 30;9:e12570. doi: 10.7717/peerj.12570 (PMC8641482; doi:10.7717/peerj.12570)

A

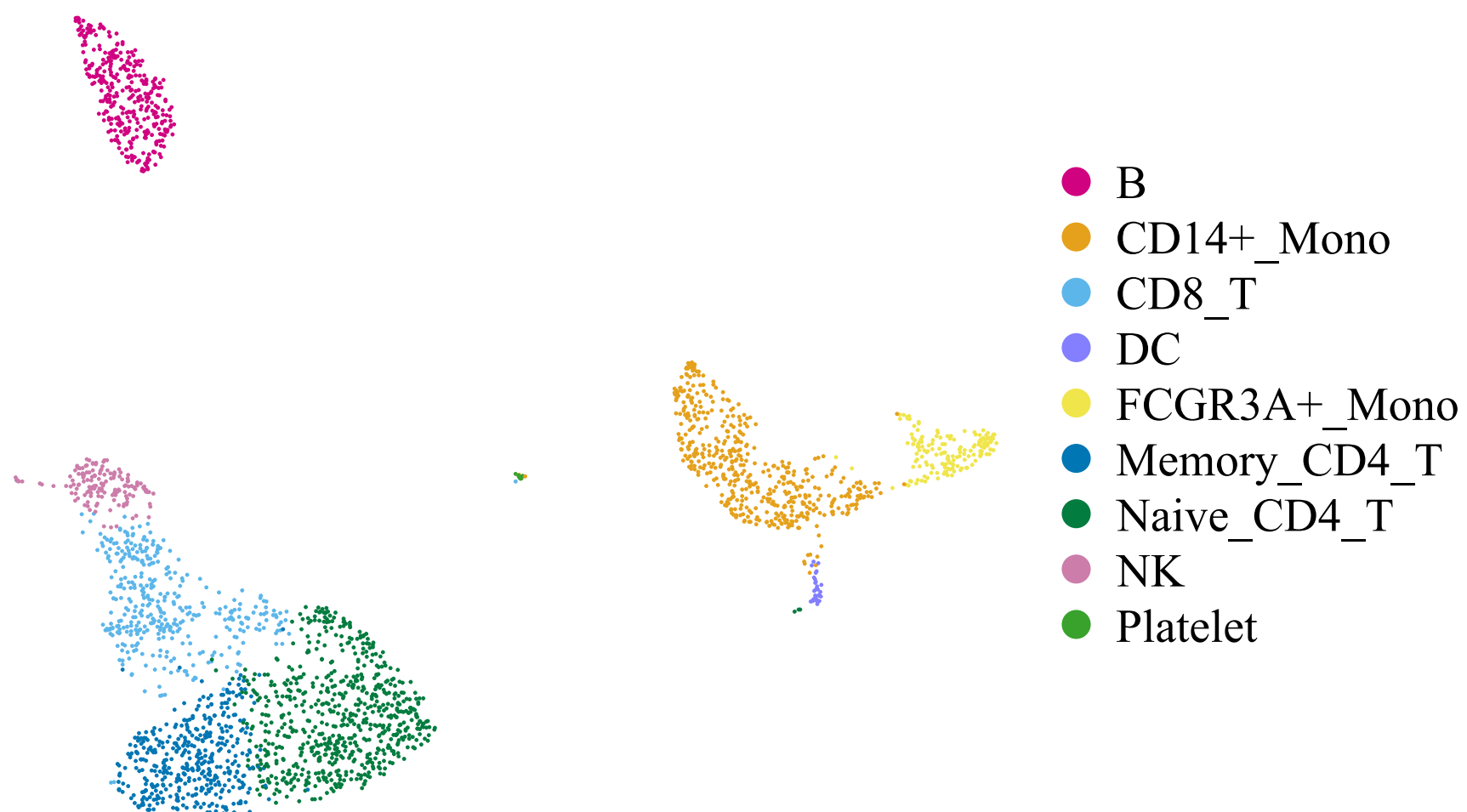

B

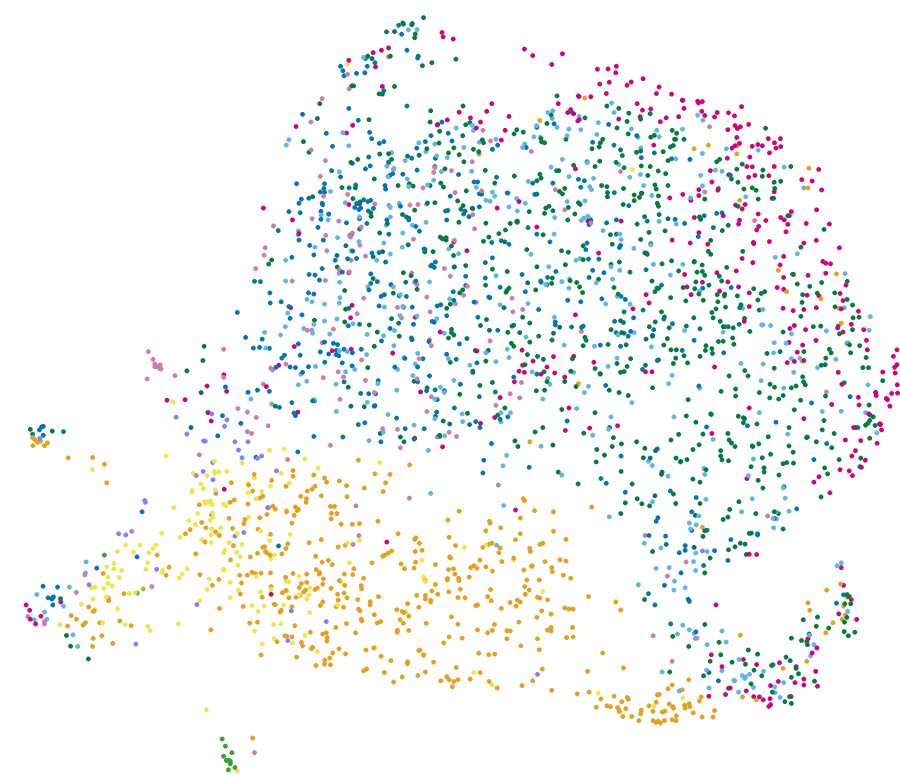

C

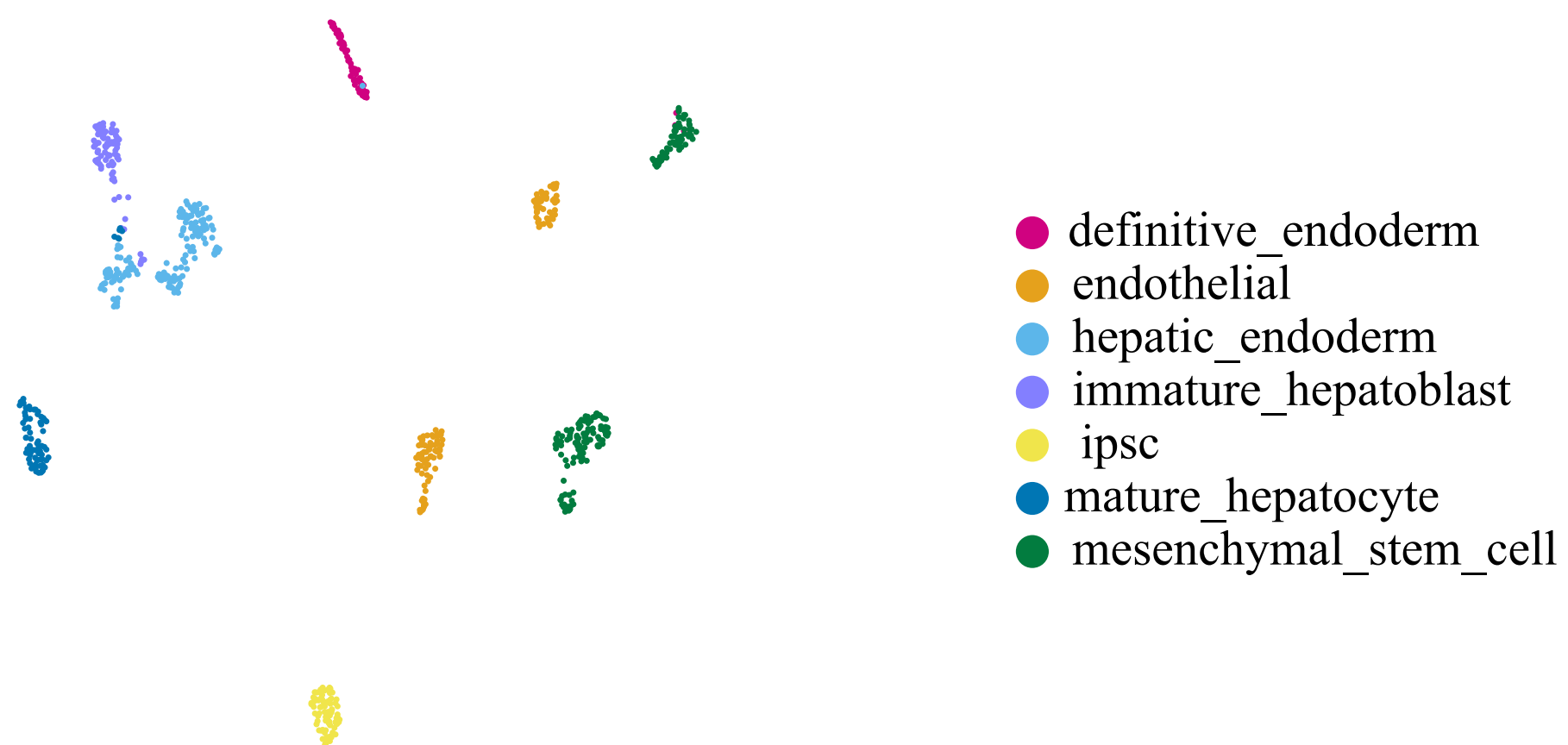

D

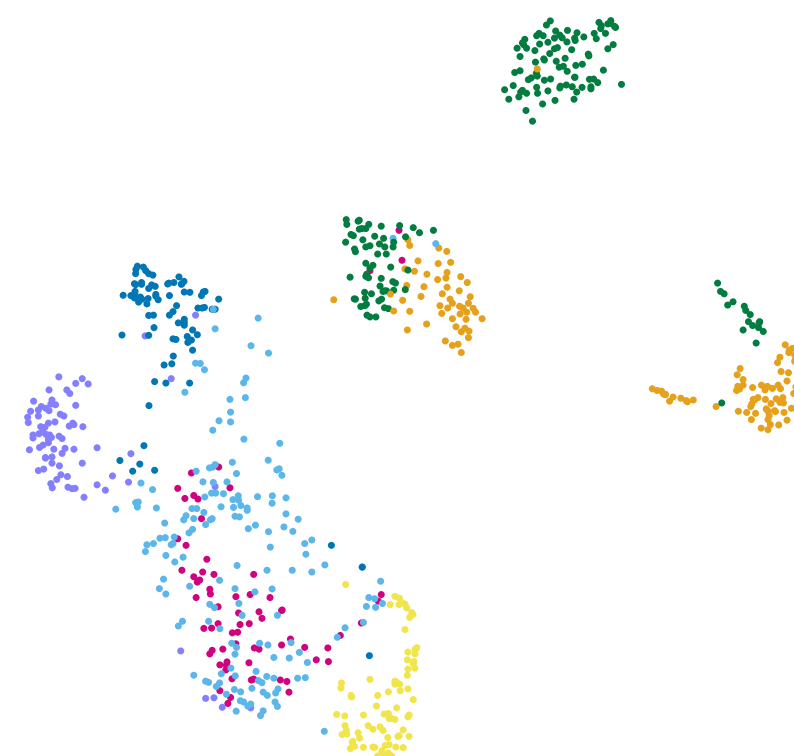

E

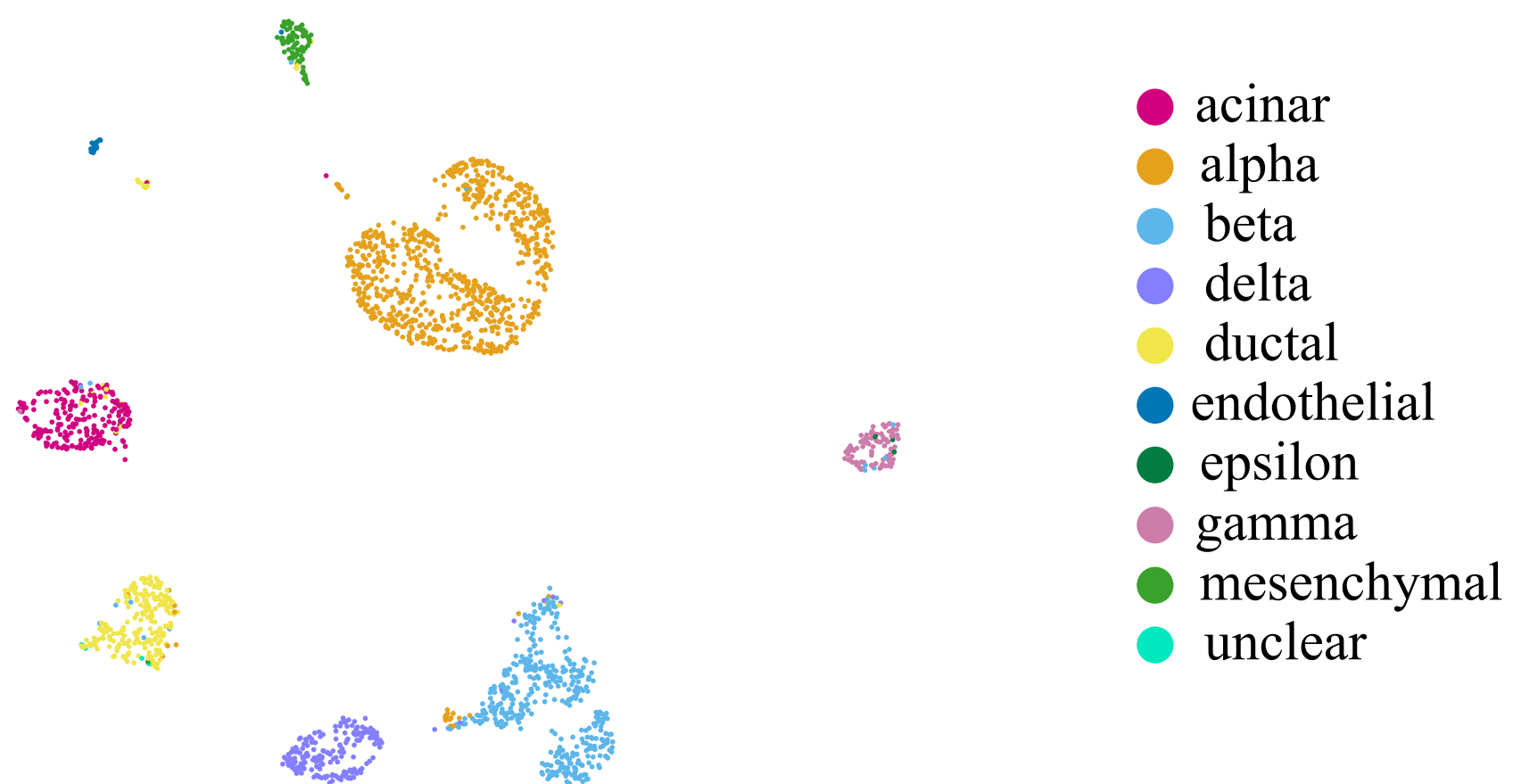

F

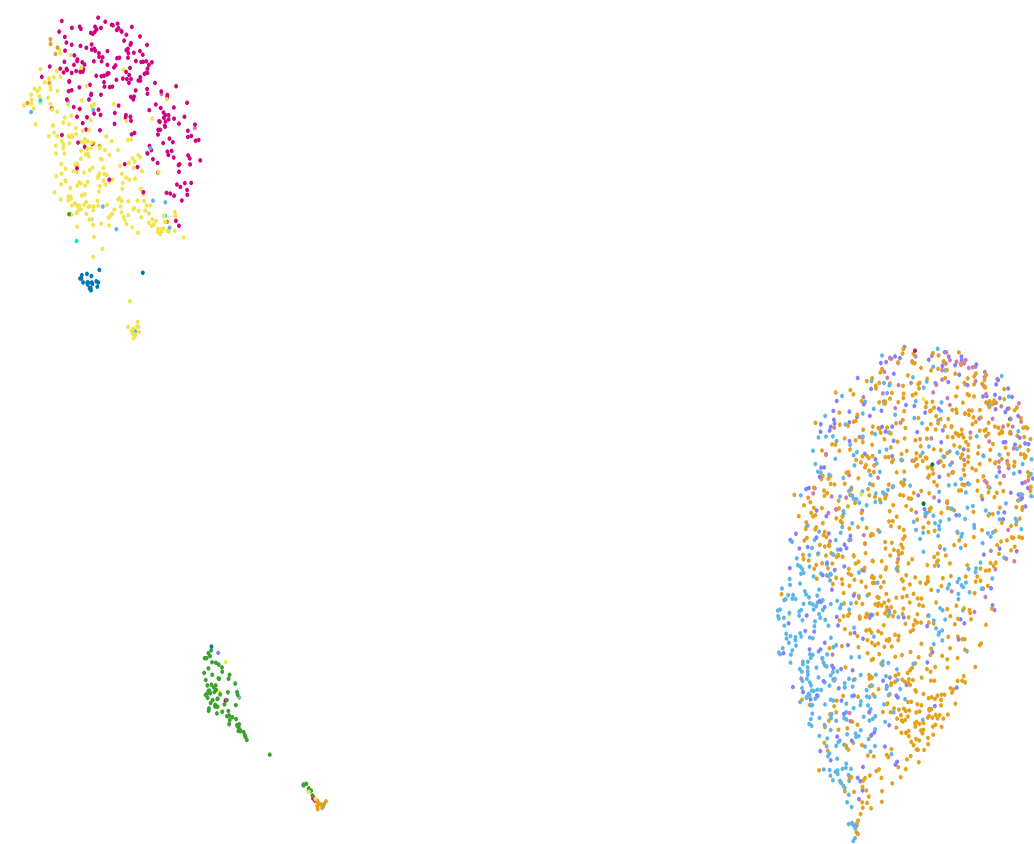

Supplement: Supplemental Information 1 — (A, C, E) The GEM clustering results of the PBMC, liver, and pancreas data sets. (B, D, F) The Reactome-based FEM clustering results of the PBMC, liver, and pancreas data sets. There are only 5,741 unique genes in the Reactome gene set. The results showed that many cells in the PBMC and pancreas datasets could not be separated (Table S1 for <!–[if !msEquation]–> <!–[if !vml]–> <!–[endif]–> <!–[endif]–> score). [file peerj-09-12570-s001.pdf]
